# The Brief Symptom Inventory-9 (BSI-9): Psychometric properties among Irish college students

**DOI:** 10.1192/j.eurpsy.2025.1486

**Published:** 2025-08-26

**Authors:** C. A. Lewis, F. Houghton

**Affiliations:** 1 Psychology, Leeds Trinity University, Leeds, United Kingdom; 2 Department of Applied Social Science, Technological University of the Shannon, Limerick, Ireland

## Abstract

**Introduction:**

The Brief Symptom Inventory-9 (BSI-9) is a recently developed short (9-item) self-report scale measuring distress. It has three Subscales measuring Anxiety, Depression, and Somatisation. The BSI-9 is based on longer scales (BSI-18, BSI-53, and SCL-90) and was developed as a brief screening tool.

**Objectives:**

The present study examined the generalisability of the original foundational research undertaken in Germany. The present study examined the psychometric properties of the BSI-9 among a sample of Irish college students.

**Methods:**

A sample of 763 Irish college students completed the BSI-9, and a further 18 students completed the BSI-9 on two occasions, separated by four weeks, as part of a more extensive study. Factor analyses and reliability analyses were carried out.

**Results:**

The BSI-9 scale and the three Subscales measuring Somatisation, Anxiety, and Depression were each found to have appropriate factor loadings, satisfactory levels of internal consistency, and satisfactory levels of temporal stability across four weeks. These findings are consistent with those reported in the original foundational research in Germany.

**Conclusions:**

Although the sample size was small and restricted to students only, the present study does provide evidence for the reliability of the BSI-9 among a sample of Irish college students. Future work is now required to extend this work to examine the convergent validity of the BSI-9 among other English-speaking samples.

**Disclosure of Interest:**

None Declared

